# Semiparametric Modeling of Daily Ammonia Levels in Naturally Ventilated Caged-Egg Facilities

**DOI:** 10.1371/journal.pone.0147135

**Published:** 2016-01-26

**Authors:** Diana María Gutiérrez-Zapata, Luis Fernando Galeano-Vasco, Mario Fernando Cerón-Muñoz

**Affiliations:** 1 Facultad de Ciencias Exactas y Naturales, Universidad de Antioquia, Medellín, Antioquia, Colombia; 2 Facultad de Ciencias Agrarias, Universidad de Antioquia, Medellín, Antioquia, Colombia; 3 Grupo de Investigación en Genética, Mejoramiento y Modelación Animal (GaMMA), Universidad de Antioquia, Medellín, Antioquia, Colombia; Columbia University, UNITED STATES

## Abstract

Ammonia concentration (AMC) in poultry facilities varies depending on different environmental conditions and management; however, this is a relatively unexplored subject in Colombia (South America). The objective of this study was to model daily AMC variations in a naturally ventilated caged-egg facility using generalized additive models. Four sensor nodes were used to record AMC, temperature, relative humidity and wind speed on a daily basis, with 10 minute intervals for 12 weeks. The following variables were included in the model: Heat index, Wind, Hour, Location, Height of the sensor to the ground level, and Period of manure accumulation. All effects included in the model were highly significant (*p*<0.001). The AMC was higher during the night and early morning when the wind was not blowing (0.0 m/s) and the heat index was extreme. The average and maximum AMC were 5.94±3.83 and 31.70 ppm, respectively. Temperatures above 25°C and humidity greater than 80% increased AMC levels. In naturally ventilated caged-egg facilities the daily variations observed in AMC primarily depend on cyclic variations of the environmental conditions and are also affected by litter handling (i.e., removal of the bedding material).

## Introduction

Poultry farming in Colombia and other tropical countries depends on naturally ventilated facilities where it is difficult to control air quality, resulting in compromised bird welfare and performance [1–7]. Ammonia (NH_3_) formation and emission are inherent to poultry production. Nitrogenous waste, such as undigested protein and uric acid, in bird excreta are precursors for NH_3_ formation by microbes [8]. Ammonia formation and volatilization is controlled by pH, temperature (T), moisture and nitrogen (N) content in excreta. Additionally, volatilization depends on factors such as the length of time excreta remains inside the facility, ventilation rates, and the level of gas concentration in the shed [6, 8–10]. Therefore, knowing the daily gas fluctuations in response to variations of the above-mentioned conditions is useful to develop in-farm strategies for controlling AMC.

Generalized additive models (GAM) have been widely used to study the effects of environmental components on human health because they are useful to model nonlinear relationships between the response variable and covariates [11–14]. GAM models only differ from generalized linear models (GLM) in that the linear predictor is replaced by a sum of unknown nonparametric smooth functions of some or all model covariates, allowing a flexible dependence expression of the response variable in the covariates [15–17]. The expression “semi-parametric model” is used when, in addition to nonparametric components (smooth functions), parametric effects (unsmoothed terms) are added to the GAM model [18].

Generalized additive models allow characterizing daily changes of air pollutants without making assumptions about the functional form of the data [11, 19]. Furthermore, its additive structure allows to include variables separately, thus increasing the explanatory power of the results [18, 20, 21]. These models have also been used for studying trends of pollutants associated with vehicle traffic [22–26] and NH_3_ concentration in water and air [27, 28], showing great explanatory power (about 80% of the variation was explained) and adjustment for non-linear relationships between weather variables and gaseous compounds. Furthermore, the model results also highlighted the importance of each effect on the response measured, showing trends in temporal and spatial variation of pollutants, thereby allowing to make inferences.

The aim of this study was to apply GAMs for modeling the curve of daily AMC in a naturally ventilated facility for caged layers.

## Materials and Methods

This study was approved by the Ethics Committee for Animal Experimentation of Universidad de Antioquia (approved on June 13, 2012).

The farm is owned by the Universidad de Antioquia and it is located in San Pedro de los Milagros (6°26’54.8” N, 75°32’35.2” W), Antioquia province (Colombia, South America). Its use in this study was approved by the Haciendas Department of the Facultad de Ciencias Agrarias. The study was conducted in a naturally ventilated shed occupied with 14406 Lohmann Brown layers between 42 and 53 weeks of age. The shed included a total of 11 modules. Three of them contained battery cages disposed in three levels (cage measurements: 58 x 34 x 23 cm; length x width x height, respectively) with 6 birds/cage (329 cm^2^ per bird). The remaining eight modules had two levels of batteries (cage measurements: 39 x 34 x 23 cm, length x width x height, respectively) with 3 birds/cage (442 cm^2^ per bird).

Two people managed the facility. The feed was produced at the farm and the daily ration was offered manually to the birds during the morning. Water was offered *ad libitum* through drinking cups and nipples. Eggs were collected two times per day. Manure accumulated in piles under the cages, and wood shavings were periodically added to manure in order to control humidity. Sick animals were separated and mortalities were collected daily.

**Fig 1:**
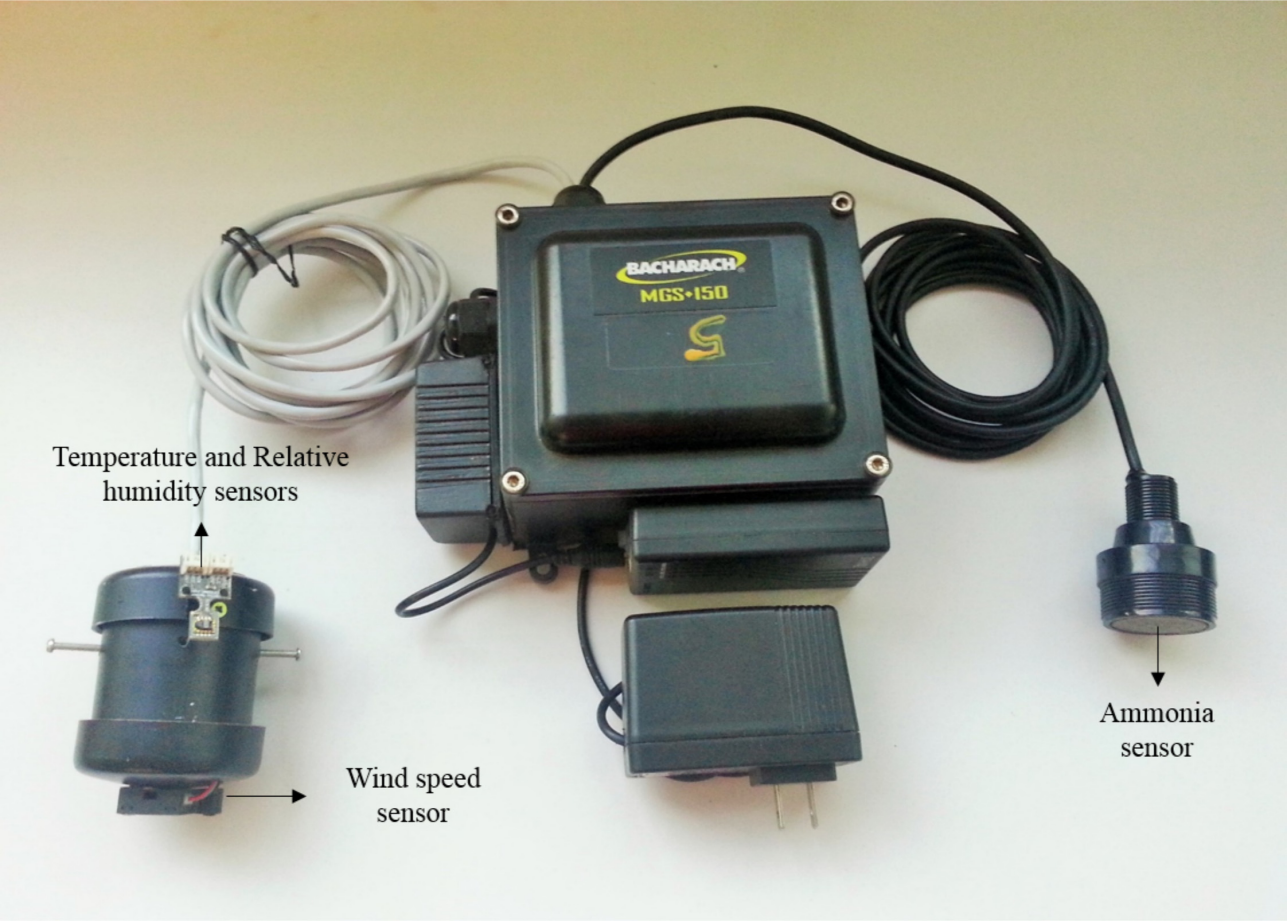
Measuring equipment (sensor node) used during the study.

Data were recorded using a multivariable monitoring system composed of four sensor nodes (Fig 1) that simultaneously measured AMC (ppm), T (°C), relative humidity (RH, %), and wind speed (WS, m/s). Sensor node specifications are in S1 Table.

The shed area was divided into 72 parts (4 m wide by 5 m long, each). Within each of these parts we were able to measure at one of four possible heights (heights of the sensor to the ground level were 1.63, 2.12, 2.78, and 3.15 m), so there were 288 possible measuring locations in total. The selection of each location was made at random; once defined, records were taken day and night at 10-minute intervals during one week.

During the 12-week period, 9708 observations from 37 days were considered in the analysis (data in S1 Dataset). Part of the information was lost due to problems with the electrical system, and some more was discarded for the analysis. Availability of information of at least two different sensors and at least 30 records/sensor per day of manure accumulation at each height was set as a requirement for the data debugging process.

The days of manure accumulation in the shed were not consecutive due to loss of information. Therefore, considering the time spacing and AMC mean values, the days of manure accumulation were grouped into eight periods. Periods of manure accumulation (PMA) were defined as follows: PMA 1 corresponds to the first 3 days of storage; PMA 2 days 4 to 6; PMA 3 days 7 to 9; PMA 4 days 23 to 25; PMA 5 days 26 to 29; PMA 6 days 29 and 30; PMA 7 days 59 and 60; and PMA 8 days 62, 64, and 65.

The combined effect of T and RH was included in the model as the heat index (HI) proposed by Schoen [29]:

$$HI=T-1.0799e^{0.03755T}[1-e^{0.0801(D-14)}]$$

where *T* is temperature (°C) and *D* is the dew point:

$$\frac{237.3*\gamma }{17.27-\gamma }$$

where:

$$\gamma =\frac{17.27*T}{237.3+T}+\ln(RH/100)$$

RH: percentage of relative humidity.

The GAM model used was:

$${log(E(Y}_{jklmnp}))=\alpha +G_{j}+W_{k}+H_{l}+Z_{m}+te(t_{n},i_{p})+\epsilon_{jklmnp}$$

where:

*Y*_*jklmnp*_ = Ammonia concentration~ Poi(μ)

α = Intercept

*G*_*j*_ = Fixed effect of PMA, where *j* ranges from 1 to 8 periods.

*W*_*k*_ = Fixed effect of wind, where *k* refers to absence (WS = 0.0 m/s) or presence (WS > 0.0 m/s) of wind.

*H*_*l*_ = Fixed effect of height of the sensor to the ground level, where *l* varies from 1 to 4 heights.

*Z*_*m*_ = Fixed effect of the location, with *m = 1*, *2*,*…*,*15* locations.

*te(t*_*n*_,*i*_*p*_*)* = Smooth function of the *n*-th hour of the day and *p*-th heat index.

*ϵ*_*jklmnp*_ = Residual effect.

The scale parameter of the Poisson distribution was included as unknown in the model in order to model over dispersion. Hour and Location were variables added in order to account for the effects of time and space. A cyclic cubic regression spline was used for Hour to ensure consistency between initial and final points. The Gam procedure of mgcv library of R program was used for the analysis [30]. Adjusted R^2^ and Generalized Cross Validation (GCV) were used for model selection, with the best fit corresponding to the highest R^2^ and lowest GCV.

## Results and Discussion

Both GAM and generalized linear models allow for non-constant variance structures, and errors should be approximately independent [18, 31, 32]. The residual plot (graphic not included) showed no patterns of residual distribution as they were randomly distributed around zero. Autocorrelation tests were conducted until the fifth lag, finding low correlations (maximum 0.29 in the fourth lag), indicating the absence of autocorrelation between residuals. All variables included in the model had an effect on it (*p*<0.001).

The adjusted R^2^ of the model was 41.50%, which is between 27 and 70% reported elsewhere [33, 34]. Richards et al. [27] used GAM to model AMC in estuary water in Australia. Their R^2^ was 88.10%. The high fit of their model could be due to the analysis of AMC in an environment different from that of the present study. According to Seedorf and Hartung [35] it is necessary to analyze many variables to determine all process interactions to account for differences in AMC inside animal facilities.

The maximum AMC was 31.70 ppm and the adjusted mean concentration was 5.94±3.83 ppm, these values are within those reported in the literature for layers Table 1. Groot et al. [36] reported mean concentrations varying between 8 and 27.10 ppm in broilers. Alloui et al. [1] reported 16.50 and 31.50 ppm AMC for naturally ventilated broiler facilities in summer during the third and seventh weeks of age respectively, while AMC did not exceed 20 ppm in forced-ventilation systems. The differences found in all these studies can be attributed, among others, to changes in ventilation rates through the season, the ventilation system used, and differences in manure management.

**Table 1:** Ammonia concentration in laying hen facilities located in different countries and production systems.

Country/year	Type of facility/ Ventilation	Manure handling	Season	Temperature (°C)	Relative humidity (%)	AMC (ppm)
England (1998) [36]	BC		S; W	10.1^a^		11.9^a^ (29%)^b^
The Netherlands (1998) [36]	BC		S; W	9.8^a^		5.9^a^ (30%)^b^
Denmark (1998) [36]	BC		S; W	8.4^a^		6.1^a^ (39%)^b^
Germany (1998) [36]	BC		S; W	10.5^a^		1.6^a^ (27%)^b^
USA (Iowa, 2003) [37]	CC; MV	HR (BF)				47^a^
USA (Iowa, 2003) [37]	MV	MB (D)				2.7^a^
USA (Iowa, 2003) [38]	CC; MV	HR (BF)		9.4 ± 11.4^c^^,^^d^	71.0 ±12.9^c^^,^^d^	44.8 (70.4%)^b^
USA (Pennsylvania, 2003–2004) [38]	CC; MV	HR (BF)		11.1 ± 10.3^c^^,^^d^	77.1±9.2^c^^,^^d^	35.9 (56.4%)^b^
USA (Iowa, 2003) [38]	CC; MV	MB (D)		9.4 ± 11.4^c^^,^^d^	71.0±12.9^c^^,^^d^	2.80 (60.4%)^b^
USA (Pennsylvania, 2003–2004) [38]	CC; MV	MB (TW)		11.1 ± 10.3^c^^,^^d^	77.1±9.2^c^^,^^d^	5.2 (65.2%)^b^
USA (Iowa, 2006) [39]	CC; MV	HR (BF)	W	18.8–22.8^e^	41–56^e^	8–20^e^
USA (Iowa, 2006) [39]	CC; MV	HR (BF)	S	28.3–30.1^e^	46–53^e^	2–4^e^
USA (Iowa, 2006) [39]	CC; MV	MB (D)	W	22.6–27.1^e^	36–47^e^	6–8^e^
USA (Iowa, 2006) [39]	CC; MV	MB (D)	S	30–31^e^	71–73^e^	2–8^e^
USA (Iowa, 2006) [39]	CC; NV	FR (BF)	W	11.4–16.8^e^	62–69^e^	20–59^e^
USA (Iowa, 2006) [39]	CC; NV	FR (BF)	S	24–25.5^e^	62–66^e^	3–15^e^
Norway, 2008 [40]	FS; MV	FR (BF)		21.4±0.09^c^	58±2.1^c^	98.2±14.1^c^
Norway, 2008 [40]	MS; MV	(W)		16.1±0.44^c^	65±2.5^c^	32.3±6.8^c^
Norway, 2008 [40]	FC; MV	(TW)		14.5±2.01^c^		5.2±4.1^c^
China (2011) [41]	CC; NV	(D)	SP			3.27±1.42^c^
China (2011) [41]	CC; NV	(D)	S			3.13± 1.85^c^
China (2011) [41]	CC; NV	(D)	A			7.96± 3.55^c^
China (2011) [41]	CC; NV	(D)	W			9.66± 2.27^c^
China (2011) [41]	CC; NV	(D)	AN	21^a^ (12.9–31.5)^e^	69^a^ (25–95)^e^	5.97± 3.27^c^
Taiwan (2011) [42]	CC; HS	FR				4.5±2.5^c^
USA (Iowa, 2011–2012) [43]	AV	MB (1/3 D); FR (BF)		23.4±0.3^c^	64±3^c^	5.2±0.5^c^
USA (Midwest, 2011–213) [44]	CC; MV	MB; (3-4D)		24.6±1.9^c^	57±9^c^	4.0±2.4^c^
USA (Midwest, 2011–213) [44]	AV;MV	MB (3-4D); FR (BF)		26.7±1.1^c^	54±7^c^	6.7±5^c^
USA (Midwest, 2011–213) [44]	EC; MV	MB; (3-4D)		25.2±1.3^c^	56±9^c^	2.8±1.7^c^

BC, Battery cages; S, Summer; W, Winter; CC, Conventional cages; MV, Mechanical ventilation; HR, High-rise; (BF), Between flocks; MB, Manure belt; (D), Daily; (TW), Twice a week; NV, Natural ventilation; FR, Floor-rise; FS, Floor housing; MS, Multilevel system; (W), Weekly; FC, Furnished cages; SP, Spring; A, Autumn; AN, Annual; HS, Half-sheltered (T control by moisture); AV, Aviary house; (1/3D), One-third of the manure belt length was removed daily; (3-4D), Manure was removed every 3 to 4 days; EC, Enriched colony house.

^a^Mean

^b^Coefficient of variation

^c^Mean ± SD

^d^Daily means outside the house

^e^Range of variation

**Fig 2:**
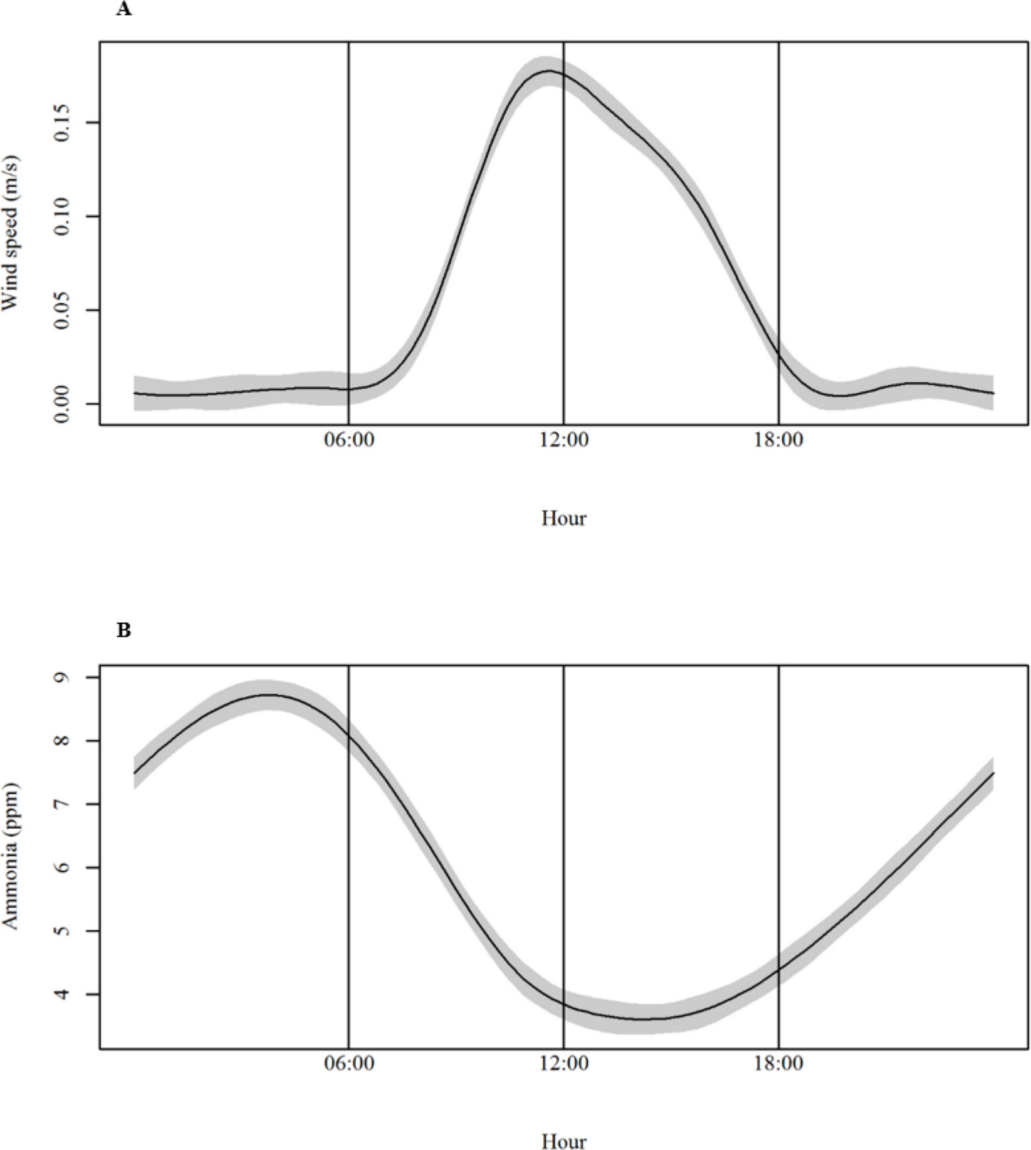
Daily variations of wind speed (A) and ammonia concentration (B).

Ammonia concentration was 0.82 ppm higher in the absence of wind. It is known that airflow helps release NH_3_ from poultry manure and contributes to its displacement. However, high speeds can also dilute NH_3_ and promote manure drying, limiting NH_3_ formation [8, 36, 45, 46]. The average AMC was high from 22:00 until the early morning, and low around noon and early afternoon. This coincided with the pattern of daily WS variations recorded in the shed, with top speeds from around noon until the evening, as shown in Fig 2.

This is similar to findings by Zhu et al. [41] who studied gas concentration and emission in naturally ventilated facilities for layers in China. They reported that gas concentration was directly influenced by environmental conditions with the highest values observed at night, when temperature and ventilation rates were lower. Different studies have shown the existence of patterns in NH_3_ concentration and emission to the atmosphere depending on season and hour of the day [41,47–49]. Harper et al. [45] observed that most NH_3_ emissions occur in the afternoon and evening, and the lowest at night. In addition, Calvet et al. [33] found higher concentrations at night and during winter compared to summertime. They attributed it to changes in ventilation rates, which were higher in the warmest hours of the day and during the summer. They also found that NH_3_ concentration and emission was negligible in the first days of the flock, increasing progressively with bird size and feed intake.

Ventilation rate of naturally ventilated facilities is affected by factors such as the number of animals in the shed, and also by the design, orientation and equipment in the facility [50–52]. Birds form living barriers that influence wind distribution within the system, changing the air flow and affecting ventilation in some areas. This affects factors such as T, RH and gasses concentration. The present study only recorded WS in the sampling locations at the time of measurement; accordingly, the dynamics of this flow is not known, so it is not possible to be precise about the extent of its effect in this study.

Both T and RH affect microbial activity so they are directly involved in NH_3_ formation and emission [53, 54]. The highest mean T was observed between 10:00 and 15:00 (fluctuating from 21 to 23°C) while the mean RH ranged from 61 to 67%. The opposite occurred during the cool hours; in the evening or early morning the mean T fluctuated between 14 and 16°C, with 83 to 85% RH.

The AMC fluctuations in time presented a sinusoidal pattern (in the form of sine and cosine functions) similar to that observed by Calvet et al. [33] and Estellés et al. [55] who modeled NH_3_ concentration and emission. In the present study, the day started with high AMC, then decreased from around 10:00 until 18:00, and increased again at night after 22:00. The AMC daily variation is attributed to diurnal cycles of T and ventilation, which also showed sinusoidal patterns.

High T generates increased NH_3_ formation and volatilization because of increased microbial degradation of uric acid and proteins. Meanwhile, ventilation helps to release NH_3_ from the manure into the environment [36].The AMC was lower in the hottest hours, probably due to the increased ventilation during those periods (Fig 2). According to several researchers, high air-exchange rates can limit AMC in a facility as a result of the acceleration of NH_3_ output (higher emissions), its dilution in the air, and because it promotes manure drying [33, 36, 41, 45, 49].

Wind speed was lower and RH increased during the night and early morning. The RH promotes increased manure moisture, which is positively related to NH_3_ production due to increased microbial degradation of uric acid. High RH values can reduce the rate of manure drying. Increasing RH from 45 to 75% generates higher AMC, while decreasing manure moisture reduces NH_3_ formation because the amount of NH_3_-N contained therein is lower [10, 33, 35, 46, 56–59].

A significant correlation between T and RH was observed (-0.69; *p*<0.05), so the HI proposed by Schoen [29] was used in order to include both effects in the model. The HI values were higher than 30°C for extreme T (> 30°C), and lower than 15°C for extreme RH (> 80%).

**Fig 3:**
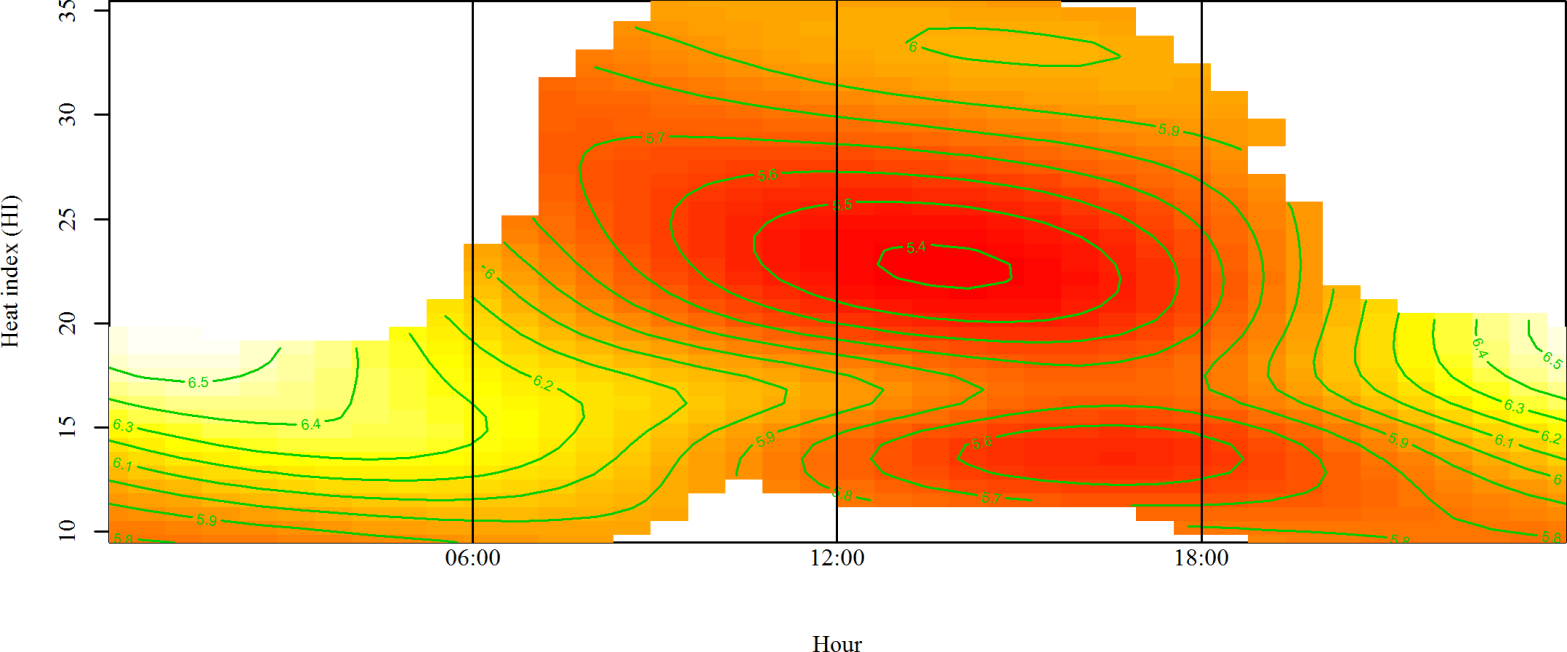
Ammonia concentration as a smooth function of hour and heat index. Ammonia level is higher in the yellow areas compared to the red ones.

Ammonia levels remained low during the day when HI was less than 15°C (Fig 3). The AMC increased from around noon until 18:00, when HI was higher than 30°C; however, the largest AMC were observed in the morning and evening hours (ends of the figure), when HI was between 15 and 20°C.

Accordingly, optimal conditions for low NH_3_ levels are close to the thermo neutral zone (between 13 and 24°C and 50 to 70% RH) [60, 61] of the birds for most of the day (06:00–18:00 hours). In this range, the lowest concentrations were observed when HI was close to 25, corresponding to T between 20 and 24°C and RH between 50 and 60%. This indicates that maintaining a suitable environment within the facility helps prevent heat stress in the birds and contributes to improved air quality due to greater control over the formation and release of harmful gases such as NH_3_. The pattern observed in Fig 3 is consistent with the cyclical variations recorded daily inside the shed.

In regard to PMA, AMC was between 1.56 and 2.15 ppm higher for PMA 2 than that observed in the other periods. However, AMC concentrations for PMAs 4 to 6 were between 0.56 and 0.58 ppm greater than those for PMA 7 and 8. These results do not agree with those reported by Neijat et al. [62], which state that when manure is compacted (as in piles) the moisture content is greater than when it is dispersed in a thin layer, with more surface contact with the air, which promotes drying and therefore reduces NH_3_ volatilization. It is important to take into account that usage of wood shavings or other material to counter humidity was low during PMA 2. Moreover, air flow into the facility has an unknown pattern, so there can be areas where the air does not flow or it is insufficient.

Removal of poultry manure from the shed should also be considered. Manure removal begins around the second month of storage. Since the removal process helps release NH_3_ the values found during this period are highly variable, regardless of the accumulation period associated with the location. As the manure removal process can take several days, some areas have few days of manure accumulation (where manure has been already removed), while other areas continue accumulating during 60 or more days. In this case air flow is the most important factor to consider as the cause of variation. Manure management consisted of forming piles under the cages and adding wood shavings to control excessive moisture. The length of the manure accumulation periods was determined by labor availability to take the manure piles out of the building, which could last two weeks or more. The maximum accumulation period recorded for an area was 75 days.

Besides the above mentioned factors it is known that NH_3_ levels are directly affected by other components such as management activities (feeding, cleaning, etc.), animal density and age, and design of the facility, among others [33, 36, 45, 49–52, 63].

According to the analysis, when the height of the sensor to the ground level increases from 1.63 to 2.12 m the NH_3_ levels decrease in 0.46 ppm. However, AMC increased 1.29 ppm at 2.78 m and then decreased 0.67 ppm at 3.15 m. According to Tinôco [61], there are three layers of air in a naturally-ventilated poultry facility: an upper layer of hot air and high NH_3_ and H_2_S concentrations, a middle layer with newly introduced air, and a lower layer of cool air which is heated on contact with the birds and receives the CO_2_ they generate. After being released from manure, NH_3_ is carried horizontally by the wind while it is dispersed in a lateral and vertical movement [54]. As NH_3_ is lighter than air, it is possible for it to quickly move to the top of the facility and therefore it should be detected in higher concentrations there. However, the pattern observed in the present study varied between heights, and only a minor difference between concentrations was observed at more than 2.12 m. According to some studies, air flow is continuous above 2 m because fewer elements are located at that height, thus air flow is not interrupted; additionally, wind speed is lower, so gas concentration tends to be constant at the top and less predictable at the bottom [64, 65]. To adequately characterize AMC variations with regard to height, it is necessary to establish the airflow dynamics within the facility, which is difficult in open sheds because of the number and complexity of the intervening variables (e.g. turbulent transport).

Even though the mean NH_3_ levels observed in this study were less than 10 ppm, at times NH_3_ values were close to the safe exposure limits for humans during an 8-hour period set by US agencies between 25 and 50 ppm [66, 67]. In poultry production there is no legal limit for exposure of birds to NH_3_; however, levels below 10 ppm are generally considered adequate for proper animal welfare and performance, with a maximum of 25 ppm at the height of the bird [68, 69]. The maximum exposure to NH_3_ levels greater than or equal to 25 ppm was 310 minutes (5 hours; between 01:00 and 06:00). Negative effects of NH_3_ on birds or staff have been widely reported [1, 3, 5, 7, 66, 70–77]. Since the effects depend on both gas concentration and time of exposure it would be necessary to further investigate whether exposure to low AMC has cumulative effects in hens during a full production cycle.

## Conclusions

Daily AMC variations depend on cyclical changes of environmental conditions (T, RH and WS). Temperature and humidity above 25°C and 80% favor increased NH_3_ levels in naturally ventilated facilities.

Generalized additive models are a suitable alternative for analyzing nonlinear relationships, such as daily NH_3_ variations and environmental factors. However, it is essential for a good fit to include the greatest possible number of factors affecting NH_3_, especially those related to the source of the gas (N, T, humidity, and pH of manure) and those associated with its release (air T and turbulent transport).

## Supporting Information

S1 Dataset This file contains the database used for the analysis presented in this work. Information of each column corresponds to: Zone of measurement (zone), date, hour, RH (humidity), T (temperature), AMC (ammonia), wind speed (windspeed), nodo, days of manure acummulation (dma), Height (height), Hour (hourofday (s)), WS (ws), PMA (pma) and HI (hi).
(CSV)

S1 Table Specifications of the monitoring system. (DOCX)
