# Supplementary material for: Semiparametric Modeling of Daily Ammonia Levels in Naturally Ventilated Caged-Egg Facilities
Source: PLoS One. 2016 Jan 26;11(1):e0147135. doi: 10.1371/journal.pone.0147135 (PMC4727935; doi:10.1371/journal.pone.0147135)
Supplement: S1 Table — (DOCX) [file pone.0147135.s002.docx]

| **Equipment** | **Operational method** | **Operational range** |
| --- | --- | --- |
| Ammonia detector | Electrochemical | 0 to 100 ppm ± 0.1 ppm |
| Temperature sensor | Band-gap sensor | -40 to 123 °C ±0.4 °C |
| Relative Humidity sensor | Capacitive sensor | 0 to 100% ±3% |
| Wind speed sensor | Differential pressure | 0-4 m/s ±5% |
